# Supplementary material for: Are carbon emissions trading and green financial instruments synergistic? -Comprehensive quantitative research based on content analysis
Source: PLoS One. 2024 Mar 7;19(3):e0298601. doi: 10.1371/journal.pone.0298601 (PMC10919601; doi:10.1371/journal.pone.0298601)
Supplement: S1 Data — (ZIP) [file pone.0298601.s001.zip › Data/地级市Z_F工作报告.zip!/╣π╢1⁄2/│▒╓▌╩╨/└┤╫╘╣1⁄2╓┌║┼í╛┬φ┐╦╩2╛▌═°í┐_2006.docx]

各位代表：

我代表市人民政府向大会作工作报告，请予审议，并请政协各位委员和其他列席人员提出意见。

一、“十五”时期的工作回顾

“十五”时期，市政府在省委、省政府和市委的正确领导下，在市人民代表大会及其常委会和市政协的监督支持下，坚持以邓小平理论、“三个代表”重要思想为指导，认真落实科学发展观和构建社会主义和谐社会的重大战略思想，不断调整充实发展思路，坚持以发展为第一要务，以结构调整为主线，以改革开放和科技进步为动力，以提高人民生活水平为根本出发点，团结全市人民奋力拼搏，保持了经济社会持续较快协调健康发展，较好地完成了“十五”计划和上次人代会确定的目标。

（一）国民经济持续较快发展

经济增长速度逐年加快。据初步核算，2005年全市实现生产总值289亿元，比上年增长12%，“十五”期间年均递增10%；人均生产总值11564元，“十五”期间年均递增9.5%，增长速度逐步接近全省的平均水平。三大产业结构调整为11.9 :53.1 :35.0。

农业基础得到巩固。去年全市农业总产值59.6亿元，比上年增长1.7%。水稻单产467公斤，比上年增产8公斤。落实最严格的耕地保护制度，粮食种植面积和总产量连年超额完成省下达考核指标。生柑、茶叶、花卉、畜牧和海水养殖等特色产业基地建设取得新进展。

特色工业快速发展。去年，全市完成工业总产值544亿元，比上年增长9.5%，“十五”期间年均递增10.4%。规模上企业达724家，比2000年增加398家，去年完成工业总产值293亿元，“十五”期间年均递增27.3%。其中规模上七大支柱行业完成工业总产值161亿元，所占比重为57%，“十五”期间年均递增28.5%。

创区域品牌和名牌名标取得丰硕成果。“十五”期间我市先后荣获“中国优秀旅游城市”、“中国婚纱晚礼服名城”、“中国瓷都”等区域性品牌；获中国驰名商标1件，中国名牌产品9项，国家免检、免验产品9项，广东省著名商标53件、名牌产品49项。

财税收入稳步增长。全市地方财政一般预算收入8.6亿元，增长21.3%，“十五”期间年均递增15.8%；各项税收总额26.9亿元，增长26.7%，“十五”期间年均递增17%。其中国税收入18.4亿元，比上年增长28.1%。地税收入8.5亿元，比上年增长23.3%。

金融运行平稳。去年底，全市金融机构本外币存款余额329亿元，各项贷款余额137亿元，“十五”期间年均分别递增15.5%和3.0%。

（二）改革开放进一步深化

各项改革稳步推进。国有企业改革继续深化，国有资产监管体系进一步完善。民营经济发展迅速，所有制结构不断优化。社会保险制度改革取得突破性进展，社会保障体系初步建立。行政体制改革稳步推进，机构编制管理得到加强，行政效率不断提高。积极探索经营城市路子，推进投融资体制和市政公用事业建设体制改革。农村二轮土地承包工作扎实推进，已通过省政府的验收。农技推广体系、动物防疫体系改革试点工作取得初步成效。粮食流通体制和国有粮食企业改革进展顺利。农村税费改革取得显著成效。计划、财政、教育、文化等方面的改革顺利推进。

对外开放水平不断提高。去年全市实现进出口总值18.9亿美元，比上年增长23.3%，“十五”期间年均递增18.2%。其中出口14.8亿美元，比上年增长25.6%，“十五”期间年均递增16.3%。陶瓷商品出口7.5亿美元，“十五”期间年均递增18.6%。利用外资总量不断扩大。去年新签利用外资项目62个，比上年增加4个；合同利用外资1.2亿美元，增长17%。“十五”期间实际利用外资4.9亿美元。新的货柜车查验场投入使用，优化了通关环境。

区域合作取得新突破。成功接待了泰国总理率领的访问团和曼谷市长率领的访问团，与曼谷市政府签署了旅游合作协议书。组织友好代表团赴曼谷市访问并正式签署协议，我市成为国内唯一与外国首都缔结友好城市的地级市。组团赴深圳学习考察并举办潮州投资环境推介会。组团赴澳门参加第十三届国际潮团联谊年会，并在年会期间举办了潮州陶瓷博览会、美食展示等活动。还承办了第九届全国“华罗庚金杯”少年数学邀请赛，举办了名瑞杯国际婚纱晚礼服设计大奖赛、中国瓷都——广东潮州（北京）陶瓷博览会、潮州首届文化旅游节等较大型活动，提高了潮州的知名度和美誉度。闽粤赣十三地市合作取得新进展。去年共接待海内外游客164万人次，比2000年增加近百万人次。旅游收入23.8亿元，比上年增长14.8%，“十五”期间年均递增18.5%。

（三）重点项目建设成果显著

固定资产投资保持较快增长。去年全市的固定资产投资97.6亿元，增长27.2%。全市46个重点项目全年完成投资39.6亿元。“十五”期间，全市固定资产投资298亿元，比“九五”期间增长88%，年均递增23.2%；工业技术改造完成投资45.7亿元，比“九五”期间增长54.4%。

基础设施建设有新进展。韩江潮州供水枢纽工程于去年9月下旬实现下闸蓄水。韩江东岸整治等一批水利项目加快施工。解决农村14.8万群众饮水特别困难问题的工程抓紧实施，饶平沿海六镇供水工程基本完成。厦深铁路潮州段工程前期工作进展顺利，潮揭高速公路潮州段动工建设。县通镇、镇通村公路工程扎实推进。2005年，全市公路总里程1939公里，公路密度为每百平方公里62.9公里，分别比“九五”期末增加143.4公里和4.6公里。“十五”期间，城乡电话交换机总容量由52.7万门增加至136万门，移动电话总容量由30万门发展至156万门。大唐电厂首期2×60万千瓦燃煤机组将于今年上半年投产，凤凰水电厂完成改造扩容工程，实施了城市电网、农村电网改造工程，供电能力明显增强。去年全市供电量33亿千瓦时，“十五”期间年均递增18.9%。

城市建设及生态建设取得显著成效。完成潮州大道、枫春路等一批道路的改造，市垃圾卫生填埋场一期交付使用，韩江北桥、桥东水厂扩建等项目进展顺利。建成滨江长廊、人民广场、文化公园等一批公共设施。扎实开展韩江、黄冈河流域环境和排污企业的综合整治，环境污染状况得到有效控制。市第一污水处理厂建成投入使用，城市生活污水处理率达到70%。林业生态建设加快，生态公益林体系和生态屏障体系基本形成。市区绿化覆盖率39.8%，环境噪声达标区覆盖率66.8%。环境质量总体保持稳定，连续五年在省环境保护责任考核中获优秀等次。以创建“国家园林城市”为目标，建立长效工作机制，强化城市环境综合整治，城市美化、净化、绿化、亮化水平不断提高，城市功能进一步完善，城市环境进一步优化，城市品位进一步提升，城市的吸引力和竞争力进一步增强。“十五”期间，市区房屋建筑竣工面积336万平方米，城市建成区面积扩大4.5平方公里。潮安、饶平县城区及城镇建设也稳步推进。

（四）人民生活水平进一步提高

城乡群众收入稳步增长。2005年末，全市居民储蓄存款余额246亿元，比上年增长15.9%，“十五”期间年均递增18.8%。市区居民人均可支配收入8946元，农民人均纯收入4260元，分别比上年增长7.1%和3.3%，“十五”期间分别递增5.3%和2.7%。

群众消费结构发生可喜变化。去年，全市社会商品零售总额103亿元，增长12.7%，“十五”期间年均递增11.2%。市区居民人均消费性支出8199元，比上年增长10%。汽车、通讯器材、商品房等大宗商品的消费持续增长。

劳动就业形势保持基本稳定。公共就业服务体系进一步完善，劳动力市场信息网络更加健全。2005年，全市新增就业岗位2.8万个，下岗失业人员再就业8480人，农村富余劳动力转移就业1.6万人。“十五”期间，全市新增就业岗位11万个，城镇登记失业率控制在2.8%以内。

社会保险扩面工作有新进展。全市参加养老保险26.3万人，比上年增长12.2%。去年全市各类社保基金收入3.8亿元，增长10.4%；支付各项待遇3.1亿元，基金收支结余6731万元。各项保险待遇实现100%社会化发放。

扶贫济困工作成效明显。全市列入最低生活保障达2.1万户、4.9万人，比上年增加7428人，低保面达到1.95%，基本做到应保尽保。市区在顺利完成三年解困规划的基础上，又制定实施了公房改危解困计划和以租赁住房补贴为主、实物配租、租金核减为辅的困难户住房保障办法。农村危房改造4个示范点已建成，209户贫困户搬入了新居。6宗大中型水库移民的生产、生活条件有所改善，解决了17条村、5066人的住房、饮水和基础设施等遗留问题。去年共有26349名农村贫困家庭子女减免了义务教育阶段的书杂费，有350名农村贫困家庭子女获得资助就读省、市技工学校。救灾救济工作进一步加强，城乡困难群众基本生活需要得到保障。新的市福利院已经竣工。

（五）精神文明建设及社会各项事业蓬勃发展

精神文明建设取得新成绩。“爱国、守法、诚信、知礼”现代公民教育活动深入开展，现代公民道德建设得到加强，以爱国主义为核心的民族精神、以改革创新为核心的时代精神和潮州人精神进一步发扬。未成年人思想道德建设得到加强和改进。富有潮州特色的群众性精神文明创建活动成效显著，城乡文明程度进一步提高，连续三届荣获“广东省文明城市”称号。

科技事业得到较大发展。“十五”期间，累计实施市级以上各类科技计划项目598项，获省科技进步奖17项，创办省级以上高新技术企业81家、民科企业94家，组建省工程技术研发中心8家，认定省专业技术创新镇试点9个。知识产权保护工作不断加强，累计专利授权3097项，居全省前列。去年，我市区域创新能力居全省第12位，企业自主创新能力居全省第9位。

教育人才工作扎实推进。“普九”成果得到巩固提高。“十五”期末，全市适龄儿童入学率98.7%，初中毛入学率105.7%，基本达到省的要求。“以县为主”的农村教育管理体制进一步完善，中小学“一费制”政策全面落实。教育信息化建设步伐加快。“十五”期间全市投入15.8亿元，新建、扩建、改建学校332所，高中、初中、小学分别新增学位2.14万个、1.89万个和3.6万个。市金山中学、高级中学新校园已建成交付使用。老区山区119所农村学校改造基本完成。市技工学校通过评估成为省重点技工学校，市卫生学校晋升为国家级重点中等职业学校。高考入围率逐年提高，去年达到84%。大力实施“人才兴市”战略，切实加强人才市场建设和人才市场信息网络建设，优化各类人才配置。重视加强专业技术人才队伍建设，进一步深化职称制度改革，不断拓宽人才评价服务领域。

文化潮州建设成效显著。古城文化旅游区规划建设加快，义兴甲历史街区、明代古城墙、广济门城楼、宋代许驸马府一期工程已修复，广济桥修复工程已进入桥上楼台亭阁的修建。潮州音乐、潮剧、潮绣艺术、潮州木雕艺术、潮州铁枝木偶和潮州剪纸艺术等项目已列入国家级非物质文化遗产公示名单，大吴泥塑、饶平布马舞通过省级非物质文化遗产项目评审。市文化艺术馆、博物馆已经动工，图书馆、档案馆等项目已完成前期工作。具有地方特色的文化活动广泛开展，群众文化生活更加丰富多彩。

人口增长得到有效控制。2005年度，全市人口出生率9.96‰，比上年下降0.35个千分点；人口自然增长率为4.42‰，比上年下降0.41个千分点。计划生育率提高至90.7%。“十五”期间，我市平均人口出生率、自然增长率分别比省下达指标低2.83个千分点和2.8个千分点。

公共卫生事业得到进一步重视。市中心医院内科大楼、潮安县人民医院、市、县疾控中心和市、县医院传染病区加紧施工。“120”医疗急救中心进一步完善配套。爱国卫生运动深入开展，城乡卫生保健水平明显提高。新型农村合作医疗逐步普及。全市参加农村合作医疗人数107.5万人，覆盖率55.9%。

各项事业全面发展。建设了全民健身广场，成功举办第十二、十三届市运会，承办广东省武术散打和围棋锦标赛。认真筹办各种群众体育活动，促进全民健身活动深入开展，竞技体育水平进一步提高。国防、民兵预备役和双拥共建工作取得明显成效，“十五”期间连续获得省“双拥模范城”称号。征兵和退役士兵安置工作得到重视，优抚政策全面落实。殡葬改革不断深化。村（居）委会换届选举工作基本完成。慈善事业和残疾人事业有新进展。新闻宣传、广播电视事业取得新成绩。民族宗教、统计、人防、气象、地震、档案、地方志等工作进一步加强。

（六）社会秩序和政务环境进一步改善

社会治安保持稳定。维稳责任制进一步落实，社会治安防控体系进一步完善。继续保持“严打”高压态势，黑恶势力犯罪、严重暴力犯罪以及“两抢两盗”等多发性侵财型犯罪得到有效遏制。深入开展扫除“黄赌毒”、打击“六合彩”和禁毒等专项斗争，净化了社会风气。法律服务、法律援助及基层调解工作成效明显，“四五”普法扎实推进。大力推进“平安和谐社区”建设，强化社会稳定基础。

经济秩序继续好转。严厉打击制假售假、偷逃税费等违法犯罪行为，进一步规范价格和收费秩序，开展反走私综合治理，强化食品安全监管，严厉查处私宰生猪行为，重视专利和知识产权保护工作，营造公平、规范、有序的市场环境。强化安全生产监管，深入开展道路交通安全、消防安全、建筑施工安全、危险化学品等专项整治，有效防止和减少事故的发生，保持全市安全生产形势的基本稳定。

行政服务水平不断提高。自觉接受人大和政协的监督，坚决执行市人大及其常委会的各项决议，认真办理人大代表的建议和政协委员的提案。去年，市政府共办理人民代表和政协委员的建议和提案164件，办结率100%。制定实施规范性文件23件。办理行政复议案件17件。“十五”期间，办理人民代表和政协委员的建议和提案558件，制定实施规范性文件111件，办理行政复议案件34件。认真贯彻实施《行政许可法》，全面推进依法行政。实行社会公示制度和听证制度，妥善处理牵涉面广、群众关注的重大事项。深入开展保持共产党员先进性教育活动和“理想、责任、能力、形象”教育，增强了公务员队伍的政治素质。廉政建设和机关效能建设取得较好成效，政务公开、要素市场建设扎实推进，行政效率和服务水平不断提高。去年，市行政服务中心各办事服务窗口共受理业务1.6万件，比上年增长42.1%，办结率99.7%。市机关效能监督投诉中心全年共受理投诉108件，办结率99%。

各位代表：“十五”期间我市经济和社会发展所取得的一切成就，是全市人民艰苦奋斗、努力拼搏的结果，也是驻潮部队、中央和省驻潮单位、各民主党派、人民团体及港澳台同胞、海外侨胞鼎力支持的结果。在这里，我谨代表潮州市人民政府表示崇高的敬意和衷心的感谢！

“十五”期间的工作使我们深刻认识到：必须坚持以科学发展观统领经济社会发展全局，才能推动我市的经济和社会全面、协调、可持续发展；必须坚持以经济工作为中心，加强基础设施建设，改善投资环境，加强对外合作，大力发展特色经济、文化经济、县域经济和港口经济，培育新的经济增长点，才能加快发展步伐；必须坚持体制创新和科技创新，增强竞争活力，优化产业结构，才能提高经济增长质量和效益；必须坚持依法行政，切实加强经济和社会管理，才能为物质文明、政治文明和精神文明建设创造良好的环境；必须坚持以人为本的理念，认真解决关系人民群众切身利益的突出问题，让广大人民群众真正享受改革开放和现代化建设的成果，才能加快构建和谐社会步伐，实现全面建设小康社会。

在肯定成绩的同时，我们也看到当前经济和社会发展中存在的突出问题：一是经济结构不够合理，产业结构不够优化，经济运行质量较低。工业经济总量不大，重化工业比重较小，企业自主创新能力不强，产品附加值较低，经济效益不高。二是生产要素制约经济发展。电力供应紧张，原材料价格上涨，石油气价格居高不下，客观上增加了生产成本，影响工业生产的效益。工业发展用地供求矛盾突出，影响现有企业扩大生产规模和好项目的引进。一些地方的工业用地规划滞后，违法租地无序发展，由此产生的环境压力日益突出。三是农民增收难度较大。农资价格涨势仍未得到有效抑制，城乡发展不够协调，转移农村富余劳动力步伐缓慢，影响农民增收的不利因素增多。四是市场经济秩序和经济规范管理仍存在很多薄弱环节，假冒伪劣、偷逃税费的现象屡禁不止，安全生产形势依然严峻。五是与经济发展密切相关的社会保障、文化建设等各项社会事业有待进一步加强。六是社会治安形势仍然较为严峻。各类侵财型犯罪的发案率仍处高位，个别地方仍然存在影响社会稳定的因素，维护稳定的任务仍然比较艰巨。对于这些问题，我们必须认真面对并努力加以解决。

二、“十一五”时期的奋斗目标和主要任务

“十一五”时期是我市全面建设小康社会进程中承先启后的关键时期。我们要落实市委关于制定“十一五”规划的建议，进一步认清形势，抓住机遇，开拓创新，推动经济社会科学发展、和谐发展，全面开创我市改革开放和现代化建设新局面。

“十一五”时期我市经济社会发展的指导思想是：以邓小平理论和“三个代表”重要思想为指导，以科学发展观统领经济社会发展全局，围绕把潮州建设成为充满活力、富有魅力、文明和谐、富裕安康的广东“东大门”的目标，转变发展观念，创新发展模式，提高发展质量，坚持以人为本，坚持抓好发展这一第一要务，优化经济结构，转变增长方式，加强自主创新，深化改革开放，加强和谐社会建设，把经济社会发展转入全面协调可持续发展的轨道，为全面建设小康社会、率先基本实现社会主义现代化打下更坚实的基础。

“十一五”时期我市经济社会发展的目标是：全市生产总值每年平均（下同）增长12%左右，人均生产总值增长11%，至2010年三次产业的比例调整为6.5：57.5：36，财政收入增长13%，固定资产投资增长18%，外贸出口增长12%，社会消费品零售总额增长13%，人口自然增长率控制在5.92‰以内，市区居民人均可支配收入增长7%，农民人均纯收入增长5%，单位生产总值能耗下降2.5%。2010年耕地保有量4.87万公顷，新型农村合作医疗覆盖率80%以上，城镇职工参加基本养老保险人数和医疗保险人数分别达到40万人、15万人，主要污染物排放总量明显减少，经济增长质量和效益明显提高，增长方式进一步转变，资源利用效率显著提高；经济结构更趋合理，自主创新能力、产业竞争力和综合实力明显增强，城乡和区域协调发展取得新突破；对外开放上新水平，内外源型经济协调发展；社会事业全面发展，精神文明建设不断加强；生态环境明显改善，生活环境更加优美，人与自然和谐发展，可持续发展能力增强；就业形势保持稳定，社会保障体系进一步健全；社会治安和安全生产状况进一步好转，社会更加和谐，人民生活水平明显提高。

围绕上述指导思想和发展目标，必须全面贯彻落实科学发展观，坚持科学发展、优化结构、提高水平、全面推进的工作方针，紧紧抓住“自主创新、品牌带动、市场开拓、港口建设、人才兴市”五个发展战略重点；做到更加注重转变经济增长方式，更加注重提高自主创新能力，更加注重促进城乡区域协调发展，更加注重深化改革开放，更加注重建设资源节约型和环境友好型社会，更加注重推进和谐社会建设。

一是调整优化产业结构。切实保护基本农田，稳定粮食种植面积。大力培育茶叶、水果、水产、畜牧、花卉等地方特色产业。加快海洋综合开发。加强畜禽防疫工作。抓好农产品质量标准体系建设。推进农业产业化经营。改造提升传统支柱产业，做强做大区域性品牌，大力发展新兴高新技术产业，加快发展大型企业集团。加强港口基础设施和省级高新技术产业区建设，积极引进、发展能源、石化、钢铁、船舶修造等基础产业。推动商贸流通、交通运输和房地产业发展，进一步做大做强文化旅游产业，推动第三产业全面发展。加快国民经济和社会信息化步伐。

二是统筹城乡区域发展。稳步推进社会主义新农村建设。全面推进农村各项改革，建立农民减负增收的长效机制。加强农村基础设施建设，完善农村公共服务体系。加快发展农村教育、科技、医疗卫生和文化等事业，促进农村社会全面进步。节约和集约使用土地，启动农村小康环保行动，改善农村生产生活环境和整体面貌。大力发展县域经济，培育市场竞争力强的优势产业、优势企业和优势产品，形成具有县域特色的产业结构。加强村镇规划、建设和管理，提高城镇化的水平。

三是促进内外源经济协调发展。优化民营经济发展环境，推动民营经济大发展、大提高。继续扩大对外开放。更好地做好“侨”字文章，加强对外交流与合作。积极参与区域合作，主动承接珠三角的产业转移。加快市开发区二期工业园区、铁汫线工业园区等招商载体的规划和建设。切实转变外贸增长方式，加大市场开拓力度，积极应对各类国际贸易摩擦，提高潮货在国内外市场的占有率。

四是致力建设“生态潮州”。积极发展循环经济。全面推行清洁生产，发展环保型产业。加快建设节约型社会，建立健全有利于资源节约和合理高效利用的机制。大力推进节水、节电、节能、节地、节材。合理保护和利用水资源。加强对生态环境、土地、海洋和矿产资源的保护。编制实施生态建设规划，加强环境综合整治，推进环保基础设施建设。

五是增强自主创新能力。加快区域自主创新体系建设，建立健全科技运行、服务和投入机制，扩大科技对外交流与合作。加强重点领域、重点区域、重点产业的自主创新。引导和鼓励企业加大技术开发投入，支持企业建立各种形式的研发机构和技术中心。加强知识产权保护和技术标准工作。推进实施品牌带动战略。着力培育一批具有自主知识产权、自主品牌、主业突出、核心竞争力强的大企业。到2010年，全市高新技术产品产值180亿元，科技进步贡献率达50%。

六是大力推进基础设施建设。大力推进厦深铁路和以高速公路、县乡公路网、港口运输系统为主体的综合交通基础设施建设。积极发展大机组煤电，开发利用小水电及风电，推广能源节约和综合利用技术。加强韩江、黄冈河及水库水源保护，抓好应急水源建设，建立稳定的水资源供给体系。搞好人防工程建设，完善三防减灾体系，加强气象减灾、种植业和畜牧业减灾及防震减灾工作。

七是深化体制改革。加快政府职能转变，强化社会管理和公共服务职能。深化财政体制改革，加大财税征管力度，增加财税收入。推进投融资体制改革，大力发展和规范资本市场。继续深化国有企业改革，分类推进事业单位改革。引导民营企业推进技术、管理、体制创新。深化文化体制改革。进一步规范产权、土地、劳动力、技术等要素市场的发展。

八是促进社会和谐发展。强化政府对义务教育的保障责任。加快教育结构和布局调整，促进教育均衡发展。创新人才工作机制，加强人才资源能力建设，优化各类人才配置。加大对文化公益事业和文化基础设施建设的投入。加强潮州文化研究，重视非物质文化遗产的保护。大力发展文化产业。深化卫生体制改革，完善公共卫生体系。完善新型农村合作医疗制度，提高农村合作医疗保障水平。加强重大传染病的防控工作。积极发展体育事业，开展全民健身运动。做好人口和计划生育工作，稳定低生育水平，提高人口素质。加强妇女、儿童和老龄人工作。继续实施民心工程，加大力度解决农村“一保五难”问题。加强就业和再就业工作。健全多层次的社会保障体系。大力发展社会慈善事业。加大扶贫开发力度，改善城乡低收入困难群众的生活。不断增加城乡居民收入，推进消费的多样化。推进社会治安防控体系建设，切实加强社会治安综合治理。严厉打击刑事犯罪，维护社会稳定。进一步提高对突发事件的快速反应和处置能力。完善公共突发事件的预警和应急体系。强化安全生产监督管理。加强食品药品安全工作。

九是推进精神文明和民主法制建设。坚持用邓小平理论和“三个代表”重要思想武装全党、教育人民。深入开展“爱国、守法、诚信、知礼”现代公民教育活动。开展群众性精神文明创建活动，树立文明新风。继续推进村（居）民自治，完善政务、村务、厂务公开。全面推进依法行政，营造文明法治环境。

三、2006年的工作意见

2006年是实施“十一五”规划的开局之年。我们必须牢固树立和认真落实科学发展观，进一步转变发展观念，创新发展模式，提高发展质量，增强发展后劲，确保安全发展，为完成“十一五”规划奠定坚实的基础。

根据省委九届八次全会的精神和市委十一届八次全会的部署，2006年全市经济和社会发展的主要预期目标是：生产总值增长12%（其中：第一产业增长1%，第二产业增长14%，第三产业增长12%）；单位生产总值能耗下降3%；地方财政一般预算收入增长15%；全社会固定资产投资增长18%；外贸出口总额增长16%；社会消费品零售总额增长13%；居民消费价格涨幅控制在3%；人口自然增长率控制在5‰以内；城镇登记失业率控制在2.8%以下；市区居民人均可支配收入增长7%；农民人均纯收入增长4.5%。

实现上述目标，必须扎扎实实地抓好以下十项工作：

（一）加大创新力度，促进产业升级

加大技术改造和技术创新力度。用好科技三项费用和创新专项资金，引导企业加强科研创新工作。争取新增工程中心5家（其中省级1家）。鼓励引导符合条件的企业参与国家、省技术改造、技术创新的招投标工作。力争全年科技进步贡献率达到46.5%。切实抓好投资总额15亿元的30个重点技术改造项目。主要有三环集团“电解质陶瓷隔膜技术产业化”、创佳集团“高亮度发光二极管生产线”、广东韩江钢板有限公司“潮州韩钢工业园建设”、潮州市松发陶瓷有限公司“高级日用细瓷无铅釉料技术开发”等项目。

继续实施品牌带动战略。加快建设国家陶瓷质量检测中心、省级食品检测中心和国家日用陶瓷生产力促进中心，引导企业采用国际标准组织生产。引导有一定知名度的企业提高产品质量和技术含量，从贴牌生产逐步转向以自主品牌生产，争创国际知名品牌。没有国家名牌的行业，要力争其产品列入国家名牌名标的评价目录，为争创国家品牌创造条件。食品行业要争取列入省产业集群示范基地。

进一步壮大特色产业。陶瓷行业要着力打造陶瓷产品研发中心、制造中心和集散中心，推进国家日用陶瓷特色产业基地建设。加快广东陶瓷职业技术学院的筹建。抓早筹备参加今年的“中小企业博览会”，增强“中国瓷都”的吸引力和辐射力。服装行业要不断创新设计，利用品牌优势扩大产品的门类品种，提高自动化生产水平和质量档次，努力打造国际婚纱晚礼服设计中心、制造中心和时尚发布中心。食品行业要推广应用现代化发酵技术、生物活性提取技术等生物工程技术，促进食品工业的现代化。大力发展果蔬、水产品的速冻保鲜加工。电子行业要开发生产精密、高质、高附加值的电子器件和微电子产品，开发生产终端家电产品。塑料制品行业要积极发展抗菌塑料制品，研发生产工业、农业、建筑业用途的产品。不锈钢制品行业要加速产品的光机电一体化，提高工业、建筑、医疗等其他用途产品的比例。印刷包装行业要加快对新型食品包装物的研发，开发生产绿色包装产品，积极发展医药包装。

（二）加快农业产业化进程，切实解决“三农”问题

坚持工业反哺农业、城市支持农村和“多予少取放活”的方针，围绕农业增效、农民增收、农村稳定这条主线，按照“生产发展、生活宽裕、乡风文明、村容整洁、管理民主”的要求，协调推进农村经济和社会各项事业的发展，加快建设社会主义新农村。

认真落实各项支农惠农政策。进一步完善农村土地承包关系，加强农村财务管理，推进农村会计代理制。切实减轻农民负担，规范农村义务教育收费管理，监督五保、优抚、低保等收费，保证各项减负工作落到实处。创新扶贫开发方式，完善扶贫龙头企业与农户的联结机制，增强带动力。加强对农民转移就业的技能培训和创业培训，促进农村劳动力向二、三产业转移。

加快农业产业化。做大做强25家市级农业龙头企业，推荐2至3家企业申报省级农业龙头企业。培育生柑、橄榄、荔枝、水产等大宗农产品的流通大户。开展市级农民专业合作经济组织试点工作，力争农业合作组织有较大发展。加快茶叶、生柑、花卉、海水网箱养鱼等特色农产品标准和农艺技术规范的制定。加快建设市级农业和渔业标准化示范区。争取“潮州凤凰茶”地理标志产品得到保护，力争通过国家无公害农、渔产品认证14项，绿色食品认证8项，新增广东省名牌产品（农业类）7项。切实稳定粮食生产，确保完成全年粮食种植任务。

加强农业农村基础设施建设。加大投入，搞好城乡水利防灾减灾工程和连片旱涝保收农田建设。积极推进农业机械化。加快行政村公路水泥硬底化建设。抓好小型灌区、养殖池塘排灌系统和蓄水工程建设，积极解决农村饮水难问题。以创建生态文明村为切入点，抓好农村改水、改厕、改路、改房、改灶，改善农业农村生态环境。完善疫情应急预警和处置机制，抓好重大动植物疫病的防控工作。

（三）挖掘文化内涵，发展特色旅游

加快古城旅游区的建设。完成牌坊街的规划设计并动工建设。争取广济桥修复工程在9月底竣工。挖掘历史文化积淀，丰富潮州旅游的文化内涵，开辟更多的生态文化游、古城文化游、工业文化游等精品线路。建设凤凰山旅游驿站，争取凤凰山——饶北山区二日游线路在国庆节开放。抓好高星级酒店项目的招商引资工作。利用广济桥、饶宗颐学术馆等项目落成的时机，举办第二届潮州文化旅游节。

积极发展文化产业。设立民间艺术保护基金，对若干濒危的民族民间文化项目进行抢救性的扶持。加强对潮州音乐、潮绣、潮州木雕等重要文化遗产项目的研究。继续深化文化体制改革，鼓励发展民营文化集团。在发展印刷、光盘等文化产业的同时，打造动漫文化品牌，带动相关产业发展。

（四）加强招商引资，开拓国内外市场

加强招商引资。充分利用各种媒体和渠道，大力宣传潮州的投资环境和特色产业优势，增强对外的影响力和吸引力。认真落实国家、省和市鼓励外商投资的优惠政策和奖励措施，调动各方招商引资的积极性。加强侨务、外事和对台工作。发挥我市招商引资顾问牵线搭桥的作用，积极开展网上招商、园区招商、项目招商、代理招商。引导外资投向高新技术产业、海洋产业、基础性交通产业、开发性农业等，进一步优化利用外资结构。

扩大外贸出口。帮助企业向国家和省争取中小企业国际市场开拓资金、民营企业出口资金、科技兴贸专项资金、反倾销应诉资金支持，提高企业的竞争力和应对风险能力。健全贸易摩擦预警机制，妥善应对贸易摩擦。充分发挥陶瓷、服装等特色产业优势，鼓励和支持企业参加各类国际经贸洽谈活动。争取更多的“广交会”摊位，特别是名优品牌摊位，让更多的企业通过这个窗口走向国际市场。积极发展加工贸易出口。尝试开展对外劳务合作及劳务输出工作。搞好潮州港口岸查验机构配套建设。加强海关、检验检疫、税务、外汇等部门的协调配合，营造良好的通关环境。

积极开拓国内市场。引导企业加强与国内其他省市的交流合作，多形式开展区域经贸合作。重视名牌名标产品的宣传推介工作，进一步扩大品牌综合效应。大力发展连锁经营、物流配送等现代流通企业。完善流通基础设施，搞好商业网点的规划和建设。切实抓好“万村千乡”市场建设工程试点工作。

（五）抓好重点项目，改善投资环境

切实抓好重点建设项目。认真贯彻落实国家宏观调控政策，进一步加快以能源、交通、水利、环保等为重点的基础设施建设，努力改善经济社会发展环境。及时了解国家产业政策及投资动态，精心筛选符合政策的项目向上争取资金支持。建立经常性的银企联系协调机制，切实为企业解决融资难问题。发挥市场配置资源的基础性作用，吸纳更多社会资金投入重点项目建设。继续配合搞好潮州供水枢纽工程建设，配合大唐电厂做好三、四号机组和风能发电项目前期工作，配合做好厦深铁路的前期工作，配合加快潮揭、汕梅高速公路潮州段建设，搞好省道335线、231线潮州段改建工程，加快500千伏潮州输变电工程前期工作等。

搞好工业园区建设。要规划建设好各类工业园区，进一步拓展企业发展空间，促进产业结构调整和土地资源的集约利用。重视发展港口经济，整合潮州港口资源，不断完善配套设施。加强宣传推介工作，争取国家级、省级的大项目布点潮州港区，争取国内外大型企业集团的石化、能源和机械装备制造项目到港区落户。加快铁汫线工业园区和市开发区二期工业园B区的规划建设，提高产业集聚水平，增强经济发展后劲。

（六）建设生态潮州，提高城市品位

进一步完善城市功能。扎实推进“创园”工作，争取明年通过国家验收。做好《潮州市城市总体规划》修编工作，加紧编制《潮州市近期建设规划》、《潮州港重化工业园综合市政规划》等。加快韩江北桥建设和桥东水厂扩建工程。建设仙洲岛堤围。加快三利溪整治工程进度和市垃圾卫生填埋场的配套项目建设。建设绿榕南路、改造潮州大道北段、改造市区排污系统等工程要尽快动工。抓紧城市供水配套建设及老管网改造。抓好西湖公园整治。加快城区通信、供电等管网统一改造工程建设。用两年时间，实现主干道管线落地、路面柏油化目标。进一步落实城市管理目标责任制，强化综合执法，依法取缔违章建筑，认真解决占道经营及“脏乱差”问题。

推进生态潮州建设。大力发展循环经济，推进资源综合利用和循环利用。鼓励资源再生利用，积极实施陶瓷废物回收利用等资源综合利用技术改造项目。引导和帮助企业对现有陶瓷窑炉进行技术改造，形成一批节约型、环保型的窑炉生产线。搞好韩江、黄冈河等重要饮用水源的保护和重点污染河流的整治。加强机动车尾气污染防治工作，确保尾气达标率达到80%。以创建生态示范村为重点，搞好生态保护工作。把韩江水质保护列入政府重要议事日程，建立长效机制，切实整治污染源。继续抓好韩江两岸林分改造、生物防火林带等生态工程。加强建设项目环保审批工作，建立健全污染源监督管理系统。重视环境监管，严防重特大环境污染事故。

合理利用土地、矿产和海洋资源。继续实行最严格的耕地保护制度，坚决遏制低水平重复建设和无序圈占土地现象。严禁毁田烧砖行为，坚决关闭并拆除实心粘土砖厂。加强土地资源的整合利用。重视矿产资源管理和地质环境保护工作，坚决查处乱采滥挖等破坏矿产资源行为。全面实施海洋环境评价和陆源污染物排放总量控制，保护和改善海洋生态环境。

（七）加强社会管理，构建和谐社会

严格控制人口增长。进一步做好人口与计划生育工作，完善基层基础建设，落实层级动态管理责任制，认真开展“镇（街道）无政策外多孩”、“村（社区）无政策外出生”活动，切实解决出生人口性别比不合理问题。建立健全依法行政、利益导向、流动人口管理、综合治理四个机制，力争把全市人口出生率、自然增长率等控制在省下达的指标以内，使全市人口计生工作达到全省中上水平。

继续整治经济秩序。进一步规范经济管理，促进公平竞争，提高经济运行质量。坚决打击制售假冒伪劣商品的违法犯罪活动。加强知识产权保护，严肃查处商业欺诈，治理商业贿赂。提高食品生产企业的市场准入门槛，取缔非法小作坊和无证照黑窝点，严厉打击使用非食品原料、添加剂或有毒有害物质生产食品的违法行为。打击虚假违法广告、非法行医和商贸领域的欺诈行为。开展药品、医疗器械和保健品专项整治。认真整顿税收和非税收费的征收管理环境。做好停业整顿地方金融机构退出市场工作。

积极维护社会稳定。进一步加强信访工作，及时发现和化解社会矛盾，完善群体性事件的预警机制和调处机制。全面推进“平安和谐社区”建设。继续保持对各类刑事犯罪的严打高压态势，深入开展打击“两抢一盗”等多发性犯罪专项斗争。深入开展禁毒专项行动。综合整治“六合彩”等赌博行为。加强对娱乐场所等特殊行业的管理。进一步落实维稳和社会治安综合治理责任制，构建群防群治网络，增强预防、控制和发现、打击违法犯罪的能力。

高度重视安全生产。抓好重大危险源监控和重大隐患整治。推行安全生产行政问责制和激励机制，狠抓责任的落实。加强安全生产监管队伍建设，提高依法监管水平。在危险化学品、建筑、特种设备、交通运输、非煤矿山、用电和公众密集场所等方面实施专项综合治理。加强全市安全生产应急救援体系建设。搞好安全生产岗位资格和安全技能培训。

切实加强应急管理。认真编制市总体应急预案、专项预案、部门预案、地方预案、企事业单位预案和重大活动应急预案。建立集中统一、坚强有力的指挥机构和应急管理队伍。加强应急物资生产、储备和避难场所的建设。普及遇灾自救和互救常识，提高全社会应对突发公共事件的能力。

（八）健全社会保障，改善人民生活

做好就业和再就业工作。继续做好农村富余劳动力转移和下岗失业人员的再就业工作。进一步完善和落实小额贷款、税费减免等再就业优惠政策。多渠道增加就业岗位，加强职业技能培训和创业培训。全市力争新开发就业岗位3万个以上，转移农村富余劳动力2.5万人。

强化社会保险扩面征缴。完善养老、失业、工伤和医疗保险制度，加快信息化服务平台建设。以上规模、上档次民营企业为重点，抓好扩面征缴工作。加快建立以失地农民为重点的农村保险制度。积极推进企业退休人员社会化管理。

完善社会救助体系。落实城乡最低生活保障制度，切实做到应保尽保。加快农村敬老院建设步伐，保障五保对象的供养。认真解决困难群众看病难、打官司难、住房难、子女入学难等问题。认真做好农村救灾和特困户救助工作。深入推进新型农村合作医疗制度改革，争取普及率达到60%以上。

推进实施饮水、安居等民心工程。积极筹措资金，加强项目建设和管理，确保14.8万人饮水解困任务按期完成。继续抓好韩西、韩东连片供水工程建设。年底前实现饶平沿海片通水。抓好第二批8个市级领导示范点工作，带动全市农村危房改造，力争今年改造危房户3500户。全面推行租赁补贴，进一步解决市区双困户的住房困难。

（九）重视精神文明，发展各项事业

推进精神文明建设。深入开展“爱国、守法、诚信、知礼”现代公民教育活动，反对封建迷信与伪科学，丰富和发展新时期潮州人精神。积极开展“双拥共建”工作，向“全国双拥模范城”迈进。深入开展创建文明社区、文明村镇、文明单位等活动。

重视人才与教育工作。全面推进农村免费义务教育，加快农村中小学的改造。解决义务教育大班制问题。实施普通高中扩容工程，大力发展职业教育和技工教育。积极实施“人才兴市”战略，加强人才资源开发和专业技术人才队伍建设。建立有潮州特色的工艺美术人才评价机制，壮大工艺美术人才队伍。

加强公共卫生工作。抓好市、县疾控中心实验室建设，完善镇级疾控组织和计免规范化门诊。加强疫情监测，防制传染性疾病的暴发流行。继续抓好市中心医院内科大楼、潮州市妇女儿童医院、潮安县人民医院建设，完成潮州卫校的迁址工作。强化卫生监督和医疗质量管理。改善农村医疗卫生条件，扩大社区卫生服务覆盖面。

发展文化体育等各项事业。抓好非物质文化遗产普查工作，落实重点文化遗产项目保护措施。加快六大文化设施和公共文化服务体系的建设。组织文化下乡活动，鼓励和发展民办文化，活跃城乡群众文化生活。加快西湖山全民健身广场等设施建设，组团参加第十二届省运会，办好第一届老年人运动会。扎实做好拥军优属工作。进一步发展广播电视、地震、气象、市志、档案、民族宗教、残疾人等各项社会事业。

（十）转变机关职能，提高行政效率

加强财税征管，建立公共财政。强化税收征管，加大依法征收力度，依法组织收入，做到应收尽收。深化“收支两条线”管理，清理和规范行政事业性收费。落实非税收入征收管理责任制考核，加强对政府资源占用收益收入、垄断性经营收入的征收管理。积极推进部门综合预算。深化国库集中支付、部门预算、政府采购、财政支出绩效评价等改革。扩大市级国库集中支付改革试点范围，稳步推进县级国库集中支付改革。加强对政府采购的监督和管理。

坚持依法行政，自觉接受监督。深化行政审批制度改革，规范审批程序。认真贯彻实施《全面推进依法行政实施纲要》，落实行政执法责任制和政府规范性文件统一审查制度。探索推行政府法律顾问工作制度。加强行政监察和审计监督，搞好行政复议和行政执法监督。推进电子政务建设和政务、事务公开。自觉接受人大和政协的监督，坚决执行市人大及其常委会的各项决议，认真办理人大代表的建议和政协委员的提案。虚心听取群众意见，认真解决群众关心的热点难点问题。

切实转变职能，提高服务效率。妥善处理好政府、市场和企业的关系，规范政府事权和投资行为。深化行政管理体制改革，合理配置职能，整合机构，强化公共服务。继续深化国有企业改革，不断完善国有资产监管体系。加强对经济运行的监测和预警，完善经济风险规避防范机制。深入开展廉政建设和反腐败斗争。深化机关效能建设，完善市行政服务中心的功能，提高行政机关的办事效率。

各位代表，今年是“十一五”规划的开局之年，做好今年的工作具有重要的意义。让我们在省委、省政府和市委的正确领导下，高举邓小平理论和“三个代表”重要思想伟大旗帜，坚持树立和落实科学发展观，同心同德，开拓进取，扎实工作，共同为建设充满活力、富有魅力、文明和谐、富裕安康的广东“东大门”而努力奋斗！
